# Deciphering the role of metal ion transport-related genes in T2D pathogenesis and immune cell infiltration via scRNA-seq and machine learning

**DOI:** 10.3389/fimmu.2024.1479166

**Published:** 2025-01-24

**Authors:** Zuhui Pu, Tony Bowei Wang, Ying Lu, Zijing Wu, Yuxian Chen, Ziqi Luo, Xinyu Wang, Lisha Mou

**Affiliations:** ^1^ Department of Endocrinology, Institute of Translational Medicine, Health Science Center, The First Affiliated Hospital of Shenzhen University, Shenzhen Second People’s Hospital, Shenzhen, Guangdong, China; ^2^ Imaging Department, The First Affiliated Hospital of Shenzhen University, Shenzhen Second People’s Hospital, Shenzhen, Guangdong, China; ^3^ Biology Department, Skidmore College, Saratoga Springs, NY, United States; ^4^ MetaLife Center, Shenzhen Institute of Translational Medicine, Shenzhen, Guangdong, China

**Keywords:** type 2 diabetes, immune cell infiltration, single-cell RNA sequencing, machine learning, protein-protein interaction, therapeutic targets

## Abstract

**Introduction:**

Type 2 diabetes (T2D) is a complex metabolic disorder with significant global health implications. Understanding the molecular mechanisms underlying T2D is crucial for developing effective therapeutic strategies. This study employs single-cell RNA sequencing (scRNA-seq) and machine learning to explore the the pathogenesis of T2D, with a particular focus on immune cell infiltration.

**Methods:**

We analyzed scRNA-seq data from islet cells of T2D and nondiabetic (ND) patients, identifying differentially expressed genes (DEGs), especially those related to metal ion transport (RMITRGs). We employed 12 machine learning algorithms to develop predictive models and assessed immune cell infiltration using single-sample gene set enrichment analysis (ssGSEA). Correlations between immune cells and key RMITRGs were investigated, and the interactions among these genes were explored through protein-protein interaction (PPI) network analysis. Additionally, we performed a detailed cell-cell communication analysis to identify significant signaling pathways in T2D.

**Results:**

Our analysis identified 1953 DEGs between T2D and ND patients, with the Stepglm[backward] plus GBM model demonstrating high predictive accuracy and identifying 13 hub RMITRGs. Twelve protein structures were predicted using AlphaFold 3, revealing potential functional conformations. We observed a strong correlation between hub RMITRGs and immune cells, and PPI network analysis revealed key interactions. Cell-cell communication analysis highlighted 16 active signaling pathways, with CXCL, MIF, and COMPLEMENT linked to immune and inflammatory responses, and WNT, KIT, LIFR, and HGF pathways uniquely activated in T2D.

**Conclusion:**

Our analysis identified genes crucial for T2D, emphasizing ion transport, signaling, and immune cell interactions. These findings suggest therapeutic potential to enhance T2D management. The identified pathways and genes provide valuable insights into the disease mechanisms and potential targets for intervention.

## Introduction

According to the International Diabetes Federation (IDF), in 2021, diabetes affected an estimated 537 million adults globally, with type 2 diabetes (T2D) making up approximately 90% of these cases (1). T2D is characterized by insulin resistance, impaired insulin secretion, and relative insulin deficiency, with a pathophysiology influenced by genetic, environmental, and lifestyle factors, leading to hyperglycemia and severe complications if not managed effectively (2). Despite standard care measures including lifestyle changes, oral hypoglycemic agents, and insulin therapy, many patients struggle to maintain optimal glycemic control, indicating a significant need for more effective treatments (3, 4). The heterogeneity of T2D contributes to the gap in personalized treatment options, underscoring the necessity to explore new strategies to enhance metabolic control and improve patient outcomes (5, 6).

Single-cell RNA sequencing (scRNA-seq) has revolutionized our ability to analyze gene expression at the cellular level (7), offering detailed insights into the cellular diversity and molecular changes that occur in T2D (8). Pancreatic islets, which comprise various cell types, including β cells, α cells, and δ cells, are pivotal in maintaining glucose homeostasis (9). Disruption in the function of these cells is a key factor in the development of T2D. Understanding the variations in gene expression and cell-cell interactions within these islets can uncover crucial pathways and regulatory networks that drive T2D.

A growing area of research focuses on the role of immune cell infiltration within pancreatic islets in T2D (10–12). Immune cells, such as macrophages and T cells, infiltrate pancreatic islets, creating a chronic inflammatory environment that worsens β-cell dysfunction. The interaction between these immune cells and islet cells plays a critical role in disease progression and can significantly impact the success of T2D management. By examining immune cell infiltration and its connection to key molecular pathways in T2D, this research aims to deepen our understanding of the disease’s immunological landscape and identify potential therapeutic targets.

The role of metal ions, such as zinc, iron, and copper, in cellular functions has emerged as a critical area of T2D research (13–15). These metal ions are essential for insulin production, secretion, and activity, with disruptions in their balance linked to metabolic disorders, including diabetes (16). Our work focuses on the novel contribution of metal ion transport-related genes (RMITRGs) in T2D pathogenesis and immune cell infiltration, which are areas that require further exploration. For instance, zinc transporters are crucial for insulin storage and release in pancreatic β-cells, and their dysfunction is associated with reduced insulin secretion in T2D (15). Similarly, the ATPase Na+/K+ transporting subunit alpha 1 (ATP1A1), vital for maintaining ion balance across cell membranes, is a promising target for therapies in T2D (17, 18). Our study explores these mechanisms further, providing a foundation for our research on the molecular underpinnings of T2D and potential therapeutic targets (19, 20).

Machine learning techniques have demonstrated considerable potential in the biomedical field, particularly in identifying predictive markers and constructing robust models for disease classification and prognosis (21). By integrating machine learning with scRNA-seq data, researchers can pinpoint hub genes and crucial regulatory networks, thereby advancing our understanding of disease mechanisms and aiding in the identification of new therapeutic targets (22, 23).

In this study, we utilized scRNA-seq along with advanced bioinformatics approaches to explore the expression and role of genes related to metal ion transport in pancreatic islet cells from T2D and nondiabetic (ND) patients. We identified differentially expressed genes (DEGs) associated with metal ion transport and constructed a protein-protein interaction (PPI) network to reveal their potential roles in T2D. Additionally, we developed predictive models using machine learning, explored the relationships between hub RMITRGs and various metabolic conditions, and assessed immune cell infiltration within islet tissues.

This study integrates single-cell transcriptomics with computational modeling, immune infiltration analysis, and network analysis, providing a comprehensive framework for uncovering the molecular underpinnings of T2D. The findings provide valuable insights into the disease mechanisms and suggest potential targets for therapeutic intervention, paving the way for more effective treatments in the future.

## Methods

### Single-cell RNA sequencing and data analysis

Single-cell RNA sequencing (ScRNA-seq) data were collected from pancreatic islet cells of 17 type 2 diabetes (T2D) patients and 27 nondiabetic (ND) individuals from PANC-DB database (24). We processed the scRNA-seq data using the Seurat package (version 4.4.0) in R. To ensure high-quality data, cells were filtered using the following thresholds: mitochondrial content less than 15%, cell count greater than 500, and gene count between 1,000 and 25,000. Highly variable genes were identified using default parameters, and data were scaled to a maximum value of 10. The data matrices were normalized (to 1,000 transcripts per cell), logged, and scaled per gene. Significant dimensions were chosen based on P values, and principal component (PC) analysis was conducted. Significant PCs were utilized for graph-based clustering. Batch effect correction was performed using the ‘RunHarmony’ function. For visualization, T-distributed stochastic neighbor embedding (T-SNE) was employed to cluster the data and visualize the major and subcellular types within the islets. Known marker genes were utilized to annotate the cell clusters (24), and the proportions of cell types by disease state were calculated.

### Identification of genes related to metal ion transport

Differentially expressed genes (DEGs) were identified by comparing T2D and ND samples across various cell types using the Wilcoxon rank-sum test and the thresholds for significance (adjusted p-value < 0.05). From the GOBP_REGULATION_OF_METAL_ION_TRANSPORT gene sets, we identified 398 genes related to metal ion transport-related genes (RMITRGs) through the Molecular Signatures Database (MSigDB) (https://www.gsea-msigdb.org/gsea/msigdb). The intersection of these RMITRGs with the identified DEGs yielded hub RMITRGs for further analysis.

### Bulk RNA data processing

T2D datasets GSE54279 (25) and GSE41762 (26) were downloaded from the GEO database using the GEOquery package in R. Dataset GSE54279, generated from Homo sapiens using the GPL6244 platform, included 128 samples from T2D patients. Dataset GSE41762, also generated from Homo sapiens via the GPL6244 platform, consisted of 77 samples (20 control and 57 T2D samples). The microarray data from these datasets were processed using the robust multiarray average (RMA) method for background correction, normalization, and probe adjustment. Batch effects were corrected using the Combat method.

### Development of 108 prediction models using machine learning

To develop predictive models for T2D, we explored 12 machine learning algorithms combined in 108 different ways. The algorithms included LASSO, Ridge, Enet, Stepglm, SVM, GlmBoost, LDA, plsRglm, GBMs, XGBoost, naive Bayes and RF models. These combinations were evaluated using area under the curve (AUC) metrics in both training and validation cohorts. The model construction used the expression data of the 49 RMITRGs identified in the scRNA-seq analysis. Seventy percent of the samples from the combined cohort (GSE54279 and GSE41762) were used for model training, and the remaining 30% were used for validation. Model performance was determined based on AUC scores, and the best-performing model was selected accordingly.

### Protein structure prediction using AlphaFold 3

To examine the structural features of hub proteins related to T2D, we employed AlphaFold 3 (27), an advanced tool for protein structure prediction. A set of hub RMITRGs associated with T2D, including ACTN4, ATF4, ATP1A1, B2M, CYBA, GNB2, HES1, PRNP, TMBIM6, TSPAN13, VMP1, and YWHAE, were selected for analysis, except for AHNAK, whose sequence length exceeded the prediction capabilities. AlphaFold 3 was configured with standard parameters to ensure accurate predictions. The primary amino acid sequences of the selected proteins were submitted to AlphaFold 3, with multiple iterations run for each protein to ensure reliable results. Confidence scores, including pLDDT and pTM, were calculated to assess the quality of the predicted structures. A pTM score above 0.5 indicated structural similarity to the true fold, while scores above 0.8 indicated high-quality predictions.

### Immune cell infiltration analysis in T2D and ND groups

The infiltration scores of immune cell types in T2D and ND groups were calculated using single-sample gene set enrichment analysis (ssGSEA) through the “gsva” R package with previously published immune cell markers (28). Visualization was performed with heatmaps using the “pheatmap” package. The correlations between immune cells and related functions were assessed with Spearman’s correlation, and the results were visualized using the “corrplot” R package and “ggplot2”. R>0.3 were considered to be positive correlation.

### Protein-protein interaction network construction

To elucidate the interactions among the hub RMITRGs and other related genes, we constructed a PPI network. This network was built using 50 genes that were closely related to our previously identified 13 hub RMITRGs, which play significant roles in metal ion transport and are differentially expressed in T2D and ND patients. These 50 genes were identified through the STRING database (https://string-db.org/) (29). The interactions within this network were visualized using Cytoscape (version 3.8.2). The cytoHubba plugin in Cytoscape was used to rank the top 10 nodes in the PPI network using five algorithms—MCC, MNC, EPC, radiality, and closeness—each providing a unique analytical perspective.

### Disease associations via a comparative toxicogenomic database

The Comparative Toxicogenomics Database (CTD) (30) was utilized to explore the interactions between the 13 hub RMITRGs and various diseases, including diabetes mellitus, T2D, glucose metabolism disorders, and metabolic diseases. Inference scores for these interactions were calculated and displayed in bar plots.

### Cell-cell communication and gene expression analysis

Cell-cell communication networks for T2D and ND patients were constructed using scRNA-seq data with the CellChat package (version 1.5.0) (31). Communication networks were based on the number of involved genes and interaction strengths across seven major cell types and subtypes. Information flow analysis identified signaling pathways active in both conditions and those uniquely active in T2D. Heatmaps were created to illustrate significant outgoing and incoming signals for specific cell groups. Receptor-ligand pair analyses were conducted to identify significant interactions associated with T2D.

### Statistical analysis

All analysis were performed in R (version 4.2.1). A P-value of less than 0.05 was considered statistically significant.

## Results

### Study workflow overview

**Figure 1:**
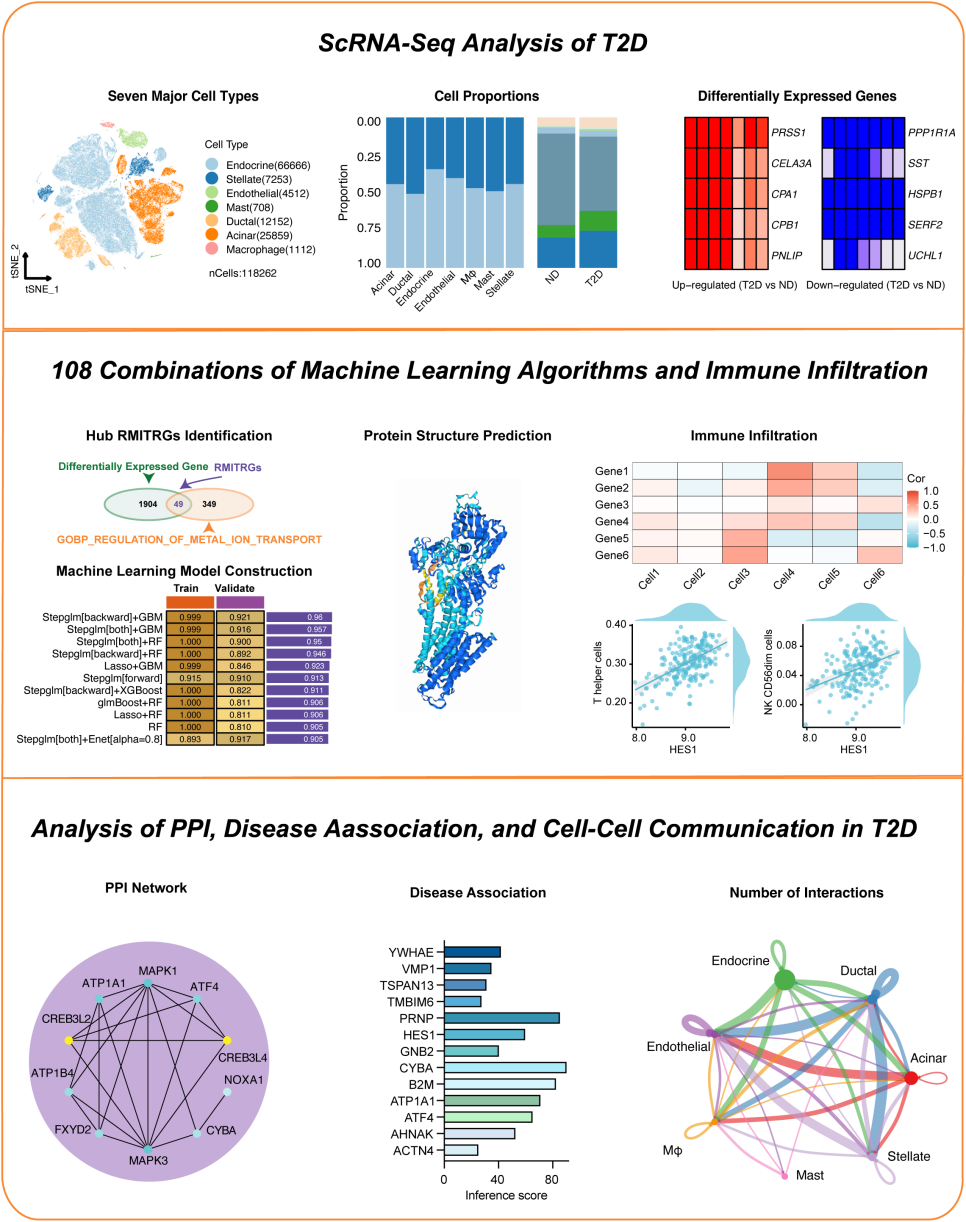
Overview of the study workflow. This figure presents a step-by-step outline of the research process.

**Figure 2:**
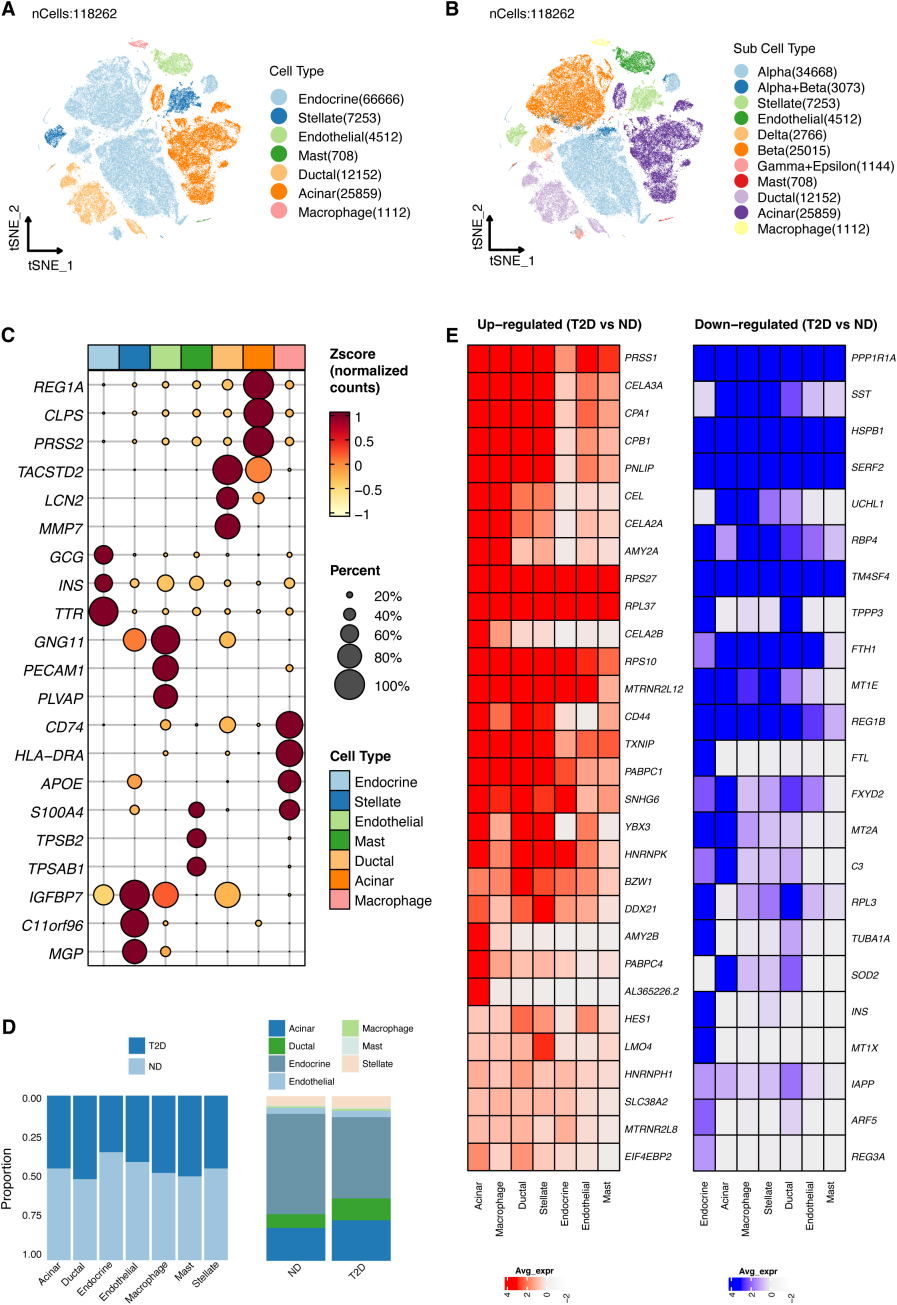
Visualization of gene expression across pancreatic islet cell types. **(A)** T-SNE plot showing the clustering of single-cell RNA-seq data from 118,262 cells isolated from 17 type 2 diabetes (T2D) patients and 27 nondiabetic (ND) individuals, identifying seven primary cell types within the islets. **(B)** Subtype analysis using T-SNE to further distinguish cell subpopulations. **(C)** Cell clusters were annotated based on established marker genes, confirming cell identity. **(D)** Comparative analysis of cell type proportions between T2D and ND samples, displayed by cell type (left) and disease status (right). **(E)** Heatmap highlighting genes with significantly altered expression in T2D patients across different cell types.

**Figure 3:**
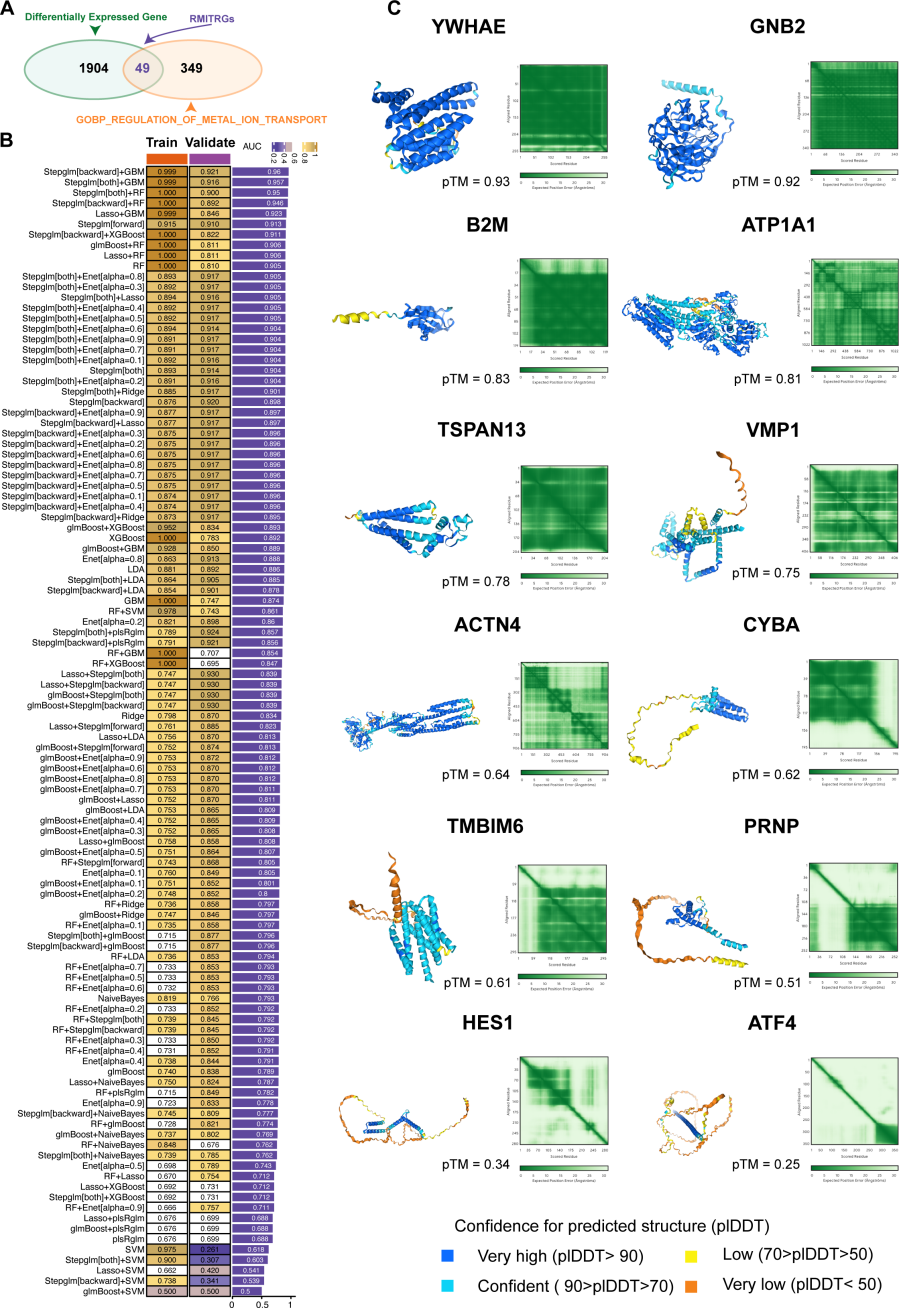
Construction and validation of predictive models using machine learning. **(A)** Identification of 1953 differentially expressed genes (DEGs) between T2D and ND samples. From the GOBP_REGULATION_OF_METAL_ION_TRANSPORT gene sets, 398 metal ion transport-related genes (RMITRGs) were identified, with 49 RMITRGs intersecting with DEGs. **(B)** Performance evaluation of 108 machine learning model combinations, with the Stepglm[backward]+GBM model achieving the highest accuracy in both training (AUC=0.999) and validation (AUC=0.921) cohorts. **(C)** Structural prediction of hub RMITRG proteins using AlphaFold 3, successfully predicting 12 out of 13 proteins, except for AHNAK due to its extensive amino acid sequence length.

**Figure 4:**
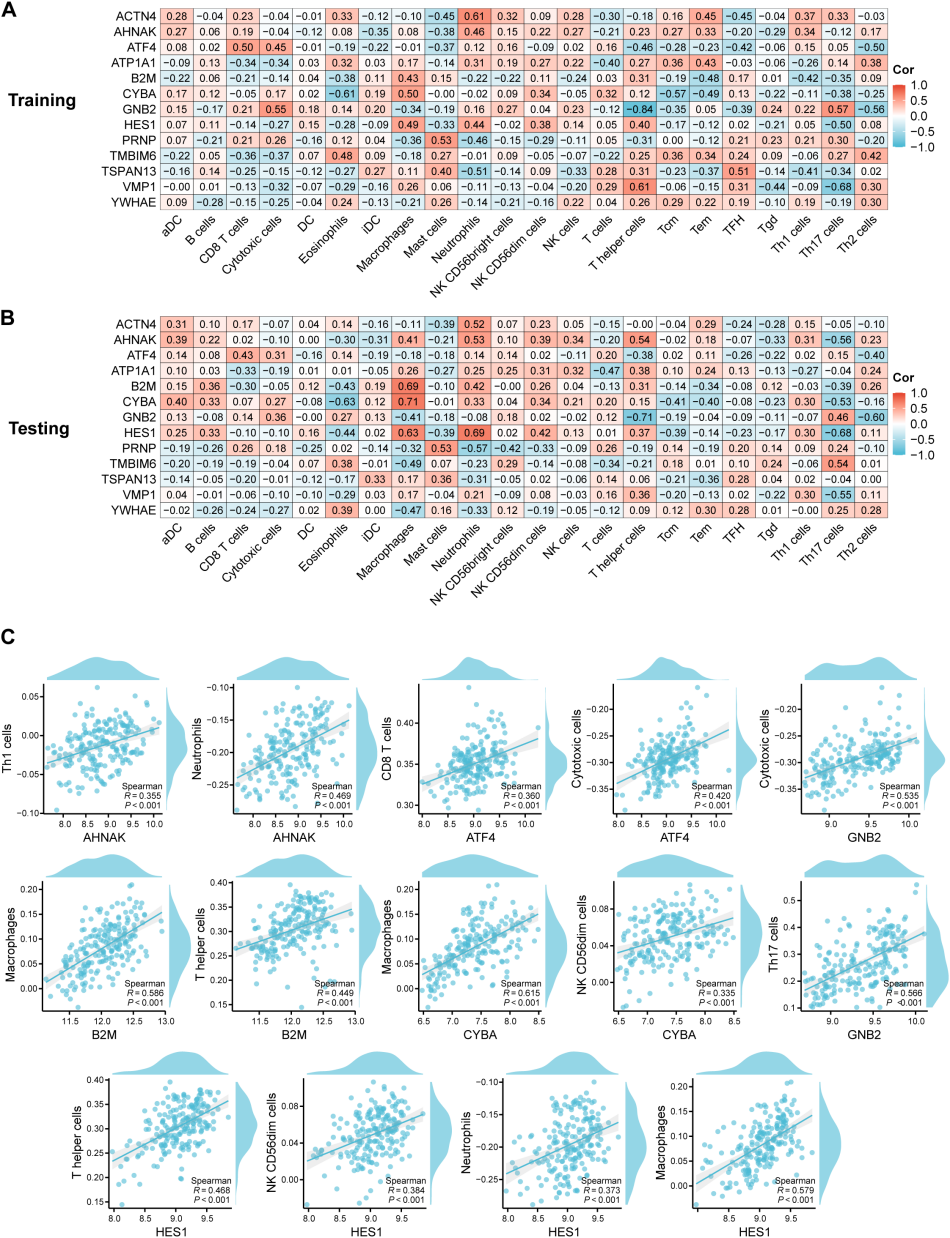
Correlation analysis between hub RMITRGs and immune cell types. **(A)** Heatmap showing the relationship between hub RMITRGs and immune cells in the training dataset. **(B)** Corresponding heatmap for the testing dataset. **(C)** Detailed correlation analysis between selected RMITRGs (AHNAK, ATF4, B2M, CYBA, GNB2, HES1) and specific immune cell types.

**Figure 5:**
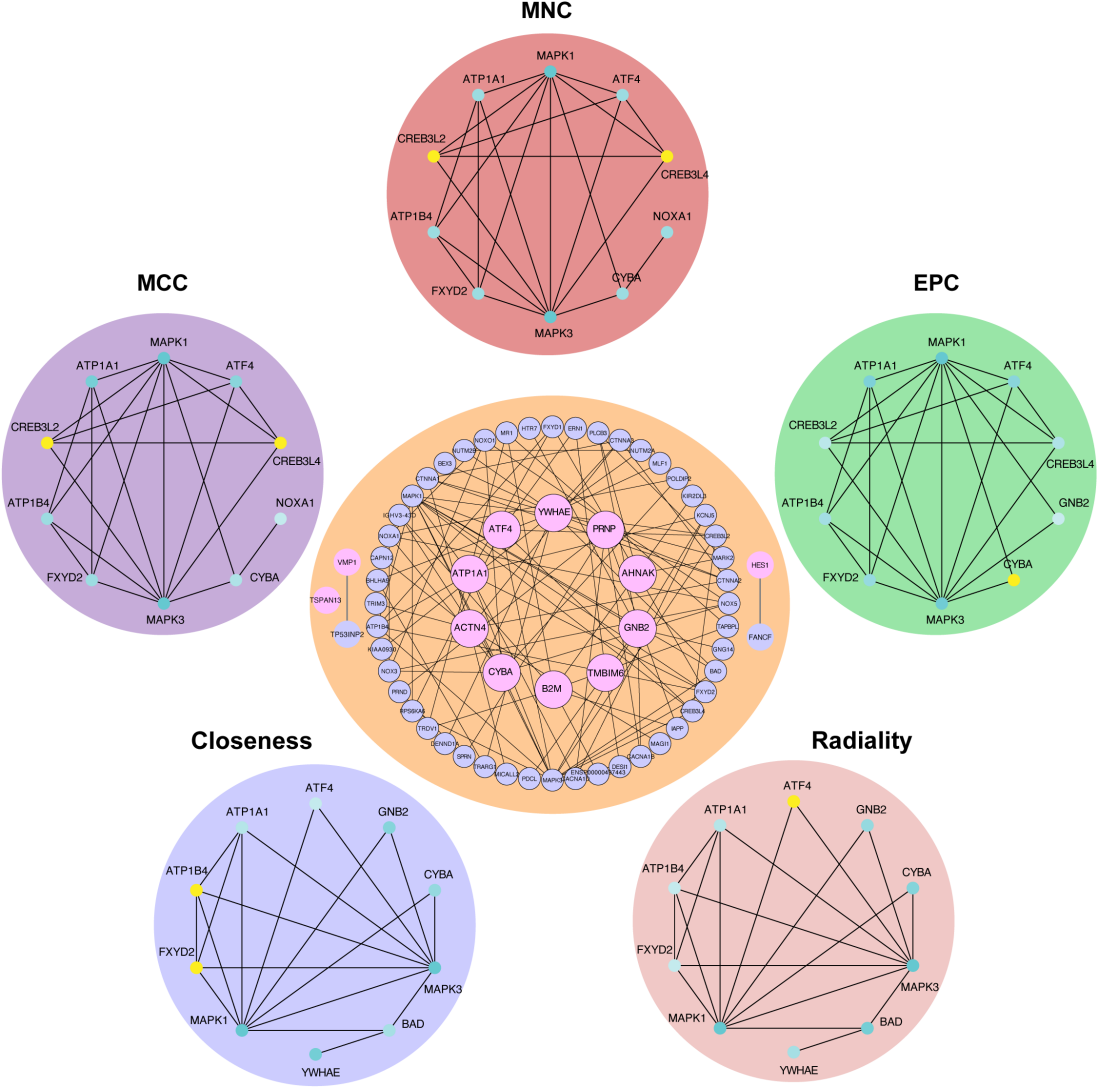
Protein−protein interaction network of hub RMITRGs. A PPI network constructed using 50 genes closely associated with the 13 hub RMITRGs, illustrating interactions among these genes. Notably, GNB2, TMBIM6, ACTN4, YWHAE, CYBA, ATF4, PRNP, ATP1A1, and B2M exhibited interconnected interactions, while TSPAN13, VMP1, and HES1 appeared independent. The top ten hub genes were determined via five distinct algorithms: MCC, MNC, EPC, radiality, and closeness.

**Figure 6:**
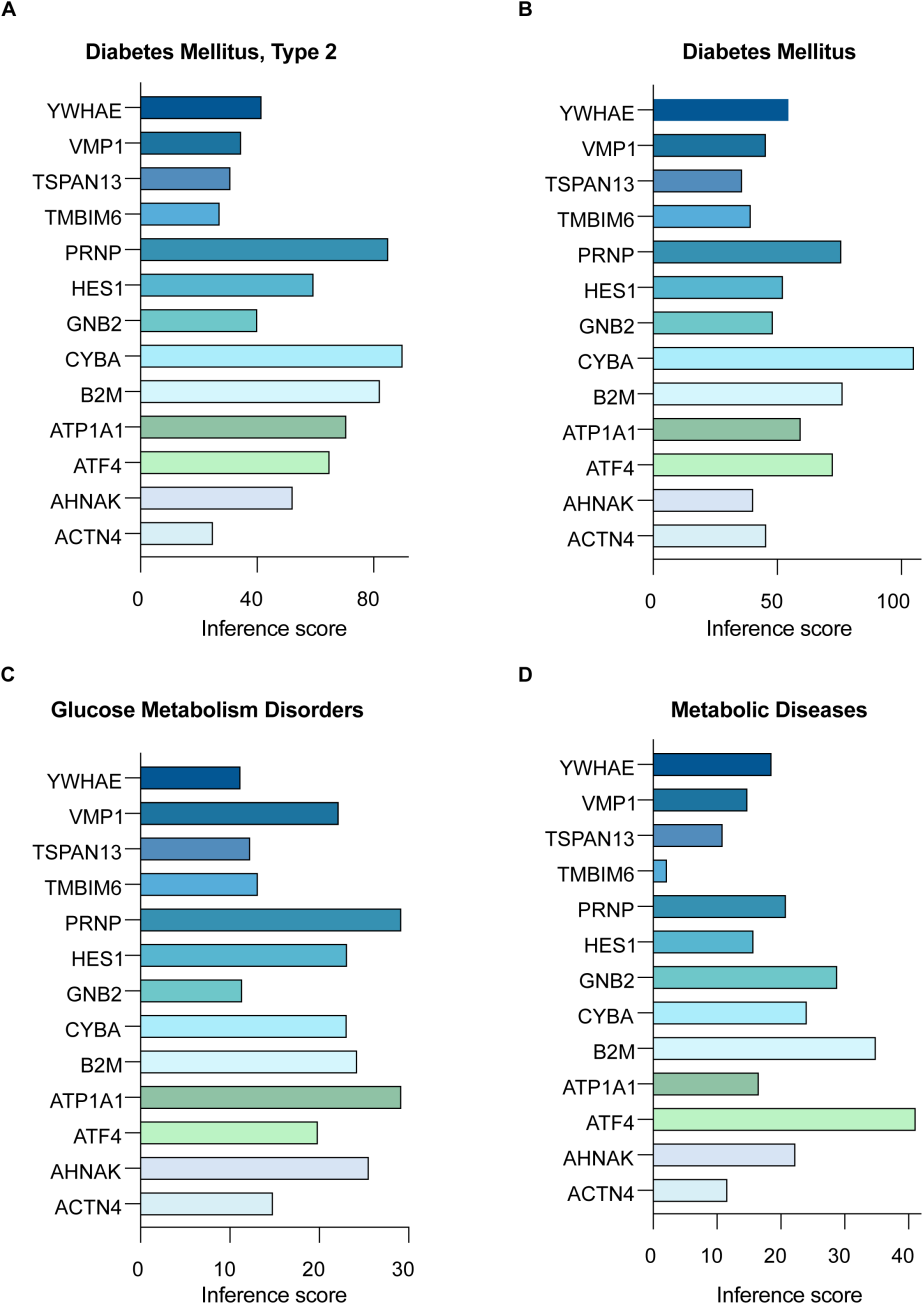
Disease associations of hub RMITRGs across multiple conditions. CTD analysis showcasing the connections between the 13 hub RMITRGs and various diseases, including **(A)** T2D mellitus, **(B)** diabetes mellitus, **(C)** glucose metabolism disorders, and **(D)** metabolic diseases, with results presented as bar plots reflecting inference scores.

**Figure 7:**
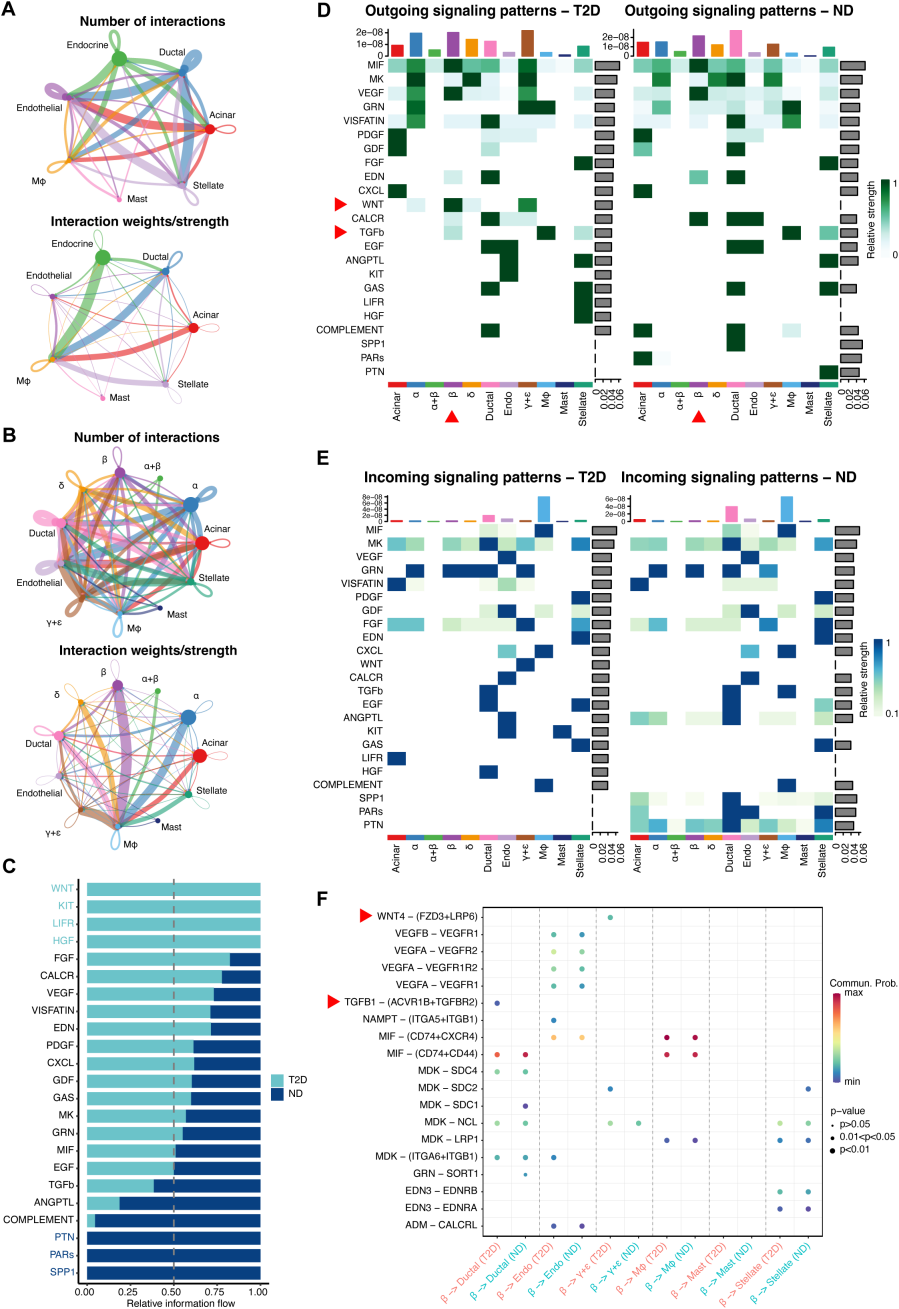
In-depth exploration of cell−cell communication and gene expression differences between T2D and ND. **(A)** Network maps displaying cell−cell communication among seven key cell types, with emphasis on the interaction strengths between endocrine cells and macrophages. **(B)** Subtype-specific communication maps, focusing on β-cell interactions with other cell subtypes, notably macrophages. **(C)** Analysis of information flow within communication networks, identifying 16 active signaling pathways, with CXCL, MIF, and COMPLEMENT linked to immunity and inflammation, and WNT, KIT, LIFR, and HGF pathways uniquely activated in T2D. **(D, E)** Heatmaps illustrating significant outgoing **(D)** and incoming **(E)** signaling pathways, particularly the WNT and TGFβ pathways in β cells of T2D patients. **(F)** Receptor−ligand pair analysis identifying Wnt4-(FZD3+LRP6) as the most prominent in T2D, primarily influencing β- to γ+δ-cell communication, with TGFB1-(ACVR1B+TGFBR2) being key in β-cell to ductal cell communication.

The study’s workflow is depicted in **Figure 1**. The investigation commenced with single-cell RNA sequencing (scRNA-seq) analysis of pancreatic islet cells from type 2 Diabetes (T2D) patients and nondiabetic (ND) individuals, revealing distinct differentially expressed genes (DEGs) and cell type distributions (**Figure 2**). Subsequent steps involved the development of machine learning models based on these DEGs, leading to the identification of key hub genes and the prediction of their protein structures (**Figure 3**). The infiltration scores of 22 immune cell types in both T2D and ND groups were then computed using single-sample gene set enrichment analysis (ssGSEA), and correlations between these immune cells and the identified hub genes were examined (**Figure 4**). A protein-protein interaction (PPI) network was constructed to explore the interactions among these hub genes (**Figure 5**), and their associations with various metabolic diseases, including T2D, were analyzed using data from the Comparative Toxicogenomics Database (**Figure 6**). Detailed analyses of cell-cell communication and gene expression highlighted significant signaling pathways and receptor-ligand pairs relevant to T2D (**Figure 7**).

### Mapping gene expression in pancreatic islet cell types

Building on our comprehensive approach to understanding T2D, We utilized scRNA-seq profiles from 118,262 cells, derived from 17 T2D patients and 27 ND individuals, to map gene expression across various pancreatic islet cell types (**Figure 2**). T-SNE analysis of the single-cell data identified seven major cell types within the islet samples, offering a comprehensive view of cell type composition (**Figure 2A**). The identified cell types included endocrine cells (GCG, INS, TTR markers), stellate cells (IGFBP7, C11orf96, MGP markers), endothelial cells (GNG11, PECAM1, PLVAP markers), mast cells (S100A4, TPSB2, TPSAB1 markers), ductal cells (TACSTD2, LNC2, MMP7 marker), acinar cells (REG1A, CLPS, PRSS2 markers), and macrophages (CD74, HLA-DRA, APOE markers). Further T-SNE analysis provided a more detailed resolution of cell identity and heterogeneity within the islets (**Figure 2B**). Clusters were annotated based on known marker genes, ensuring accurate identification of cell types, including β cells, α cells, δ cells, and other islet cell types (**Figure 2C**). We compared our cell type annotations with those from the previous study (24), highlighting the similarities and differences in cluster assignments (**Supplementary Table S1**). Analysis of cell proportions by disease state highlighted differences in cellular composition between T2D and ND samples (**Figure 2D**). A heatmap illustrated the genes significantly upregulated or downregulated in T2D patients compared to healthy controls across different cell types (**Figure 2E**). This analysis provided critical insights into the molecular changes associated with T2D, laying the groundwork for our subsequent investigations into the functional roles of these genes and their potential as biomarkers or therapeutic targets.

### Identification of DEGs and their functional roles

Continuing from our detailed mapping of gene expression in pancreatic islet cell types, we explore the molecular signatures that distinguish T2D from ND states. This led us to the identification of DEGs, which are crucial for understanding the pathophysiology of T2D. A total of 1953 DEGs were identified between β cells of T2D and ND samples (**Figure 3A**). Among these, 398 metal ion transport-related genes (RMITRGs) were identified from the GOBP_REGULATION_OF_METAL_ION_TRANSPORT gene sets. The intersection of DEGs with these RMITRGs yielded 49 RMITRGs. Research on these genes is vital as metal ions play critical roles in various cellular processes, including enzymatic activities, signal transduction, and maintaining cellular homeostasis. Disruption in metal ion transport can impact insulin secretion and sensitivity, potentially contributing to T2D pathogenesis. By identifying and analyzing these RMITRGs, we can gain a deeper understanding of the molecular mechanisms underlying T2D and uncover potential therapeutic targets to improve disease management.

### Development and validation of machine learning models

Having established the differential gene expression profiles and the significance of RMITRGs in T2D, we proceeded to leverage these discoveries in a novel direction—employing machine learning to predict T2D with greater accuracy. This approach allowed us to utilize the power of computational algorithms to decipher complex patterns within the expression data of the 49 RMITRGs identified through our scRNA-seq analysis.

To predict T2D, we explored 12 machine learning algorithms, combined in 108 different configurations, and evaluated their performance using AUC metrics in both training and validation cohorts. The expression data of 49 RMITRGs identified in the scRNA-seq analysis were used to construct the models. The combination of Stepglm[backward] and GBM algorithms produced the best results, achieving an AUC of 0.999 in the training cohort and 0.921 in the validation cohort, with an average AUC of 0.96 (**Figure 3B**). This high level of accuracy highlights the model’s robustness in distinguishing between T2D and ND states. The Stepglm[backward]+GBM model incorporated 13 genes, including ACTN4, AHNAK, ATF4, ATP1A1, B2M, CYBA, GNB2, HES1, PRNP, TMBIM6, TSPAN13, VMP1, and YWHAE, underscoring their potential as biomarkers for T2D.

The protein structures of 12 out of the 13 hub RMITRGs were successfully predicted using AlphaFold 3, except for AHNAK, whose amino acid sequence length exceeded the prediction capacity of AlphaFold 3 (**Figure 3C**). The structural predictions provided valuable insights into the potential functional conformations of these proteins. The overall predicted folds for most proteins, including YWHAE, GNB2, B2M, ATP1A1, TSPAN13, VMP1, ACTN4, CYBA, TMBIM6, and PRNP, were considered to be reliable, with pTM scores above 0.5, indicating high confidence in their predicted structures. However, pTM scores for HES1 and ATF4 were below 0.5, suggesting lower confidence and the need for further experimental validation. These results highlight the potential of combining machine learning and structural biology approaches to identify and validate key molecular players in T2D. The identified hub genes and their predicted structures offer a foundation for future functional studies and therapeutic targeting, with implications for improving T2D management.

### Correlation between hub RMITRGs and immune cells

Expanding on our exploration of the molecular landscape of T2D, we shifted our focus to the intricate relationship between hub RMITRGs and immune cell infiltration, a critical aspect of the disease’s pathogenesis. This transition allowed us to bridge the gap between genetic expression and immunological implications, providing a more comprehensive perspective of T2D’s complex dynamics.

To further investigate immune cell infiltration and function between T2D patients and ND controls, ssGSEA was used to evaluate the enrichment scores of various immune cell subsets and functions. The correlation between 13 hub RMITRGs and immune cells was analyzed in both the training (**Figure 4A**, **Supplementary Table S2**) and testing sets (**Figure 4B**, **Supplementary Table S3**), revealing strong positive correlations with immune cells for several genes, including ACTN4, AHNAK, ATF4, ATP1A1, B2M, CYBA, GNB2, HES1, PRNP, TMBIM6, TSPAN13, VMP1, and YWHAE. Specifically, AHNAK, ATF4, B2M, CYBA, GNB2, and HES1 showed strong positive correlations with at least two immune cell types (**Figure 4C**). For instance, AHNAK was strongly correlated with Th1 cells and neutrophils; ATF4 with CD8 T cells and cytotoxic cells; B2M with macrophages and T helper cells; CYBA with macrophages and NK CD56dim cells; GNB2 with cytotoxic cells and Th17 cells; and HES1 with T helper cells, NK CD56dim cells, neutrophils, and macrophages. Additionally, ACTN4, PRNP, TMBIM6, TSPAN13, and VMP1 were positively correlated with neutrophils, mast cells, eosinophils, and T helper cells, respectively (**Supplementary Figure S2**). Only ATP1A1 and YWHAE were not associated with immune cells.

These correlations provide valuable insights into the immunological aspects of T2D and suggest potential avenues for therapeutic intervention. They also highlight the need for further research to elucidate the precise mechanisms through which these hub RMITRGs influence immune cell behavior and contribute to the disease’s progression.

### PPI network analysis of hub RMITRGs

Building upon our understanding of the correlation between hub RMITRGs and immune cells, we extended our analysis to investigate the complex web of interactions that these genes may participate in within the cellular context of T2D. This led us to conduct a PPI network analysis, which is crucial for deciphering how these genes might work in concert or independently to influence disease outcomes.

The PPI network constructed for 50 genes closely related to the 13 hub RMITRGs provided insights into the molecular interactions and regulatory mechanisms involved in T2D. Within this network, GNB2, TMBIM6, ATP1A1, ACTN4, YWHAE, CYBA, ATF4, PRNP, and B2M exhibited significant mutual interactions, suggesting their central roles and potential collaborative functions in disease progression. In contrast, TSPAN13, VMP1, and HES1 did not interact with other hub RMITRGs, indicating that they might function independently or within different molecular pathways (**Figure 5**). To identify the most critical nodes within the PPI network, five analytical algorithms—MCC, MNC, EPC, radiality, and closeness—were used. These algorithms help determine the hub genes based on different aspects of network topology, such as connectivity and centrality. The top ten hub genes identified by these algorithms were intersected, revealing seven genes consistently appearing across all methods: MAPK1, MAPK3, ATP1A1, ATF4, ATP1B4, FXYD2, and CYBA (**Supplementary Figure S2**, **Supplementary Table S4**). This consensus highlights the importance of these genes in the network and their potential as key regulators in T2D.

These findings underscore the complexity of T2D at the molecular level and the potential for targeted therapies that could disrupt disease progression by modulating these critical interaction networks. The PPI network analysis not only enhances our understanding of T2D’s pathophysiology but also shed light for future research aimed at unraveling the detailed mechanisms through which these hub genes exert their effects.

### Associations between hub RMITRGs and disease conditions

Following our exploration of the PPI network and the identification of key genes with significant interactions, we proceeded to investigate the broader implications of these hub RMITRGs in the context of various disease conditions. This step was crucial for understanding the scope of their influence beyond T2D and for identifying their potential roles in other metabolic disorders.

Analysis using the Comparative Toxicogenomics Database (CTD) highlighted the relationships between the 13 hub RMITRGs and various disease conditions. Inference scores were calculated for T2D (**Figure 6A**), Diabetes Mellitus (**Figure 6B**), Glucose Metabolism Disorders (**Figure 6C**), and Metabolic Diseases (**Figure 6D**), with significant associations displayed in bar plots. These results provide valuable insights into how these genes are implicated in various metabolic diseases, emphasizing their importance in the pathology of T2D.

Specifically, five genes—ATF4, ATP1A1, B2M, CYBA, and PRNP—emerged with the highest inference scores in the context of T2D and Diabetes Mellitus, suggesting that these genes play critical roles in the molecular mechanisms underlying these conditions. For instance, beta-2-microglobulin (B2M) is integral to the immune response as a component of MHC class I molecules. Elevated levels of B2M are associated with inflammation and metabolic disorders, indicating its role in the inflammatory processes contributing to insulin resistance and β-cell dysfunction in T2D.

Furthermore, the prion protein (PRNP), traditionally linked with prion diseases, also has functions in cellular processes such as signal transduction, cell adhesion, and protection against oxidative stress. Its association with T2D suggests broader roles in maintaining cellular health under metabolic stress conditions. The analysis extended to glucose metabolism disorders and broader metabolic diseases, reinforcing the significance of these hub genes across various metabolic contexts. This consistent identification across multiple disease conditions underscores their central role in metabolic regulation and their potential as therapeutic targets.

Other hub RMITRGs, such as HES1, TMBIM6, TSPAN13, and VMP1, also showed significant associations with metabolic diseases (**Figure 6D**), highlighting the diverse molecular pathways involved in T2D. HES1, for example, is involved in the regulation of developmental processes and cell differentiation, and its association with metabolic diseases suggests a potential role in the regulation of pancreatic β-cell function and insulin secretion. TMBIM6 is known for its ability to inhibit apoptosis and regulate calcium homeostasis, possibly contributing to its protective effects on β-cells and influence on cellular stress responses in T2D. TSPAN13 and VMP1 are involved in processes like cell adhesion, signal transduction, and autophagy, which are critical for cellular maintenance and response to stress, further implicating them in T2D pathogenesis.

These findings underscore the complex and diverse characteristics of the hub RMITRGs and their potential implications in a range of metabolic conditions, providing a foundation for future research aimed at elucidating their specific roles and developing targeted therapeutic strategies.

### Cell-cell communication and gene expression analysis

Transitioning from the examination of hub RMITRGs and their association with disease conditions, we shifted our focus to the intricate realm of cell-cell communication and gene expression, which are pivotal in understanding the complex dynamics within the pancreatic islets. This analysis was crucial for elucidating the molecular dialogues that could influence T2D pathology and potentially reveal new avenues for therapeutic intervention.

A detailed investigation into cell-cell communication and gene expression differences between T2D and ND patients was conducted, focusing on critical interactions and signaling pathways that play a role in T2D pathology. Communication network maps of the seven major cell types revealed extensive interactions, particularly between endocrine cells and other islet cell types, such as endothelial cells, macrophages, stellate cells, acinar cells, and ductal cells (**Figure 7A**). The strongest interactions were observed between β cells and macrophages, indicating that macrophages may significantly influence β-cell function and survival in the T2D environment.

Further analysis of subcellular type communication revealed that β cells, the primary insulin-producing cells, engage in extensive communication with all subcell types within the islets. The strongest interactions were again noted with macrophages, emphasizing the potential role of immune cells in modulating endocrine cell behavior in the diabetic state (**Figure 7B**). This highlights the critical influence of immune cells on β-cell dysfunction in T2D.

Information flow analysis identified sixteen signaling pathways that were active in both T2D and ND conditions. Notably, three pathways—CXCL, MIF, and COMPLEMENT—are associated with immunity and inflammation, underscoring the chronic inflammatory state in T2D. In addition, four pathways—WNT, KIT, LIFR, and HGF—were uniquely activated in T2D, suggesting their role in disease progression and β-cell dysfunction (**Figure 7C**). The unique activation of these pathways in T2D provides insights into the specific molecular alterations driving the disease and highlights potential therapeutic targets.

Heatmaps were generated to illustrate the significant contributions of various signals to outgoing and incoming signals of specific cell groups, providing a visual representation of the complex communication networks within the islets. Notably, the WNT and TGFβ pathways were significantly activated in the outgoing signaling of β cells in T2D, indicating their involvement in β-cell signaling alterations and possibly in the maladaptive responses of these cells in the diabetic state (**Figures 7D, E**).

Receptor-ligand pair analysis revealed that Wnt4-(FZD3+LRP6) was the most significant pair in T2D, particularly contributing to communication between β cells and γ+δ cells. These findings suggest that Wnt4 signaling plays a crucial role in the cross-talk between β cells and other islet cell types, potentially impacting insulin secretion and overall islet function. Additionally, the TGFB1-(ACVR1B+TGFBR2) receptor-ligand pair was prominent in communication between β cells and ductal cells, further highlighting the importance of TGFβ signaling in interactions between β cells and other islet components (**Figure 7F**). This comprehensive analysis of altered cell-cell communication networks in T2D sheds light on critical pathways and interactions that may contribute to disease progression, offering potential targets for novel therapeutic strategies aimed at modulating islet cell interactions and improving β-cell function and survival in T2D.

## Discussion

Addressing the complexities of type 2 diabetes (T2D) (32) necessitates a deep understanding of the underlying molecular mechanisms. In this study, we adopted a comprehensive approach by integrating single-cell RNA sequencing (scRNA-seq), machine learning, and protein-protein interaction (PPI) network analysis to unravel these complexities. Our findings shed light on the intricate gene expression landscape, intercellular communication, and key regulatory pathways in pancreatic islet cells from T2D patients.

The scRNA-seq analysis provided significant insights, revealing pronounced alterations in the gene expression profiles of pancreatic islet cells from T2D patients compared to nondiabetic (ND) controls. T-SNE clustering exposed distinct cellular populations and highlighted shifts in the proportions of major cell types, particularly β cells, which are essential for insulin secretion. The differential gene expression analysis identified 1,953 differentially expressed genes (DEGs), offering a broad list of genes potentially implicated in T2D pathogenesis. These results emphasize the critical role of β-cell dysfunction in T2D and suggest new molecular targets for therapeutic interventions.

By integrating machine learning algorithms with scRNA-seq data, we developed predictive models for T2D. Among the 108 combinations of the 12 machine learning algorithms tested, the Stepglm[backward]+GBM model achieved the highest predictive accuracy. This model identified 13 key metal ion transport-related genes (RMITRGs), with 12 of their protein structures successfully predicted using AlphaFold 3. However, AHNAK, due to its large sequence length, could not be modeled, reflecting a limitation of current computational tools for handling very large proteins. The identified hub genes are promising biomarkers for T2D diagnosis and potential targets for novel therapies.

The construction of the PPI network revealed crucial interactions among the hub RMITRGs, spotlighting key regulatory nodes such as ATP1A1, GNB2, TMBIM6, and ACTN4. The identification of top hub genes through multiple analytical algorithms highlighted their central role within the network. Further analysis using the Comparative Toxicogenomics Database (CTD) underscored strong associations between these hub genes and various metabolic disorders, including T2D, glucose metabolism disorders, and general metabolic diseases. This underscores the broad relevance of these genes and their potential as therapeutic targets.

Our study explore the correlation between hub RMITRGs and immune cells, revealing significant interactions that offer insights into the pathogenesis of T2D. The integration of these analyses provides a comprehensive understanding of the potential roles of RMITRGs in modulating immune cell behavior and their infiltration within the islet cells. The detailed biological mechanisms of RMITRGs and immune cell interactions:

1. Biological Rationale: The role of metal ions in immune cell function is well-established (33), with ions such as zinc and iron being critical for cell signaling, redox homeostasis, and inflammation modulation. Given the central role of these ions, alterations in the expression of RMITRGs could influence the local microenvironment, impacting immune cell infiltration and activity in the islets.
2. Correlation Analysis: Our findings indicate a strong correlation between hub RMITRGs and immune cells, suggesting that the differential expression of these genes could modulate the inflammatory response in T2D. This correlation underscores the potential for RMITRGs to serve as regulatory nodes in the immune cell-mediated inflammatory process within the islets.
3. Mechanistic Relationship: The mechanistic relationship between RMITRG expression and immune cell infiltration is multifaceted: (1) Altered Metal Ion Homeostasis: Changes in RMITRG expression could disrupt metal ion homeostasis (34), influencing the redox balance and modulating inflammatory responses within the islets (35). (2) Inflammatory Signaling Pathways: The differential expression of RMITRGs could activate inflammatory signaling pathways, such as NF-κB, central to immune cell activation and cytokine production (36). (3) Direct Impact and Implications for T2D Pathogenesis: Immune cell infiltration, influenced by the expression of RMITRGs, plays a pivotal role in β-cell function and the progression of T2D. Our findings suggest that changes in RMITRG expression could directly impact β-cell function by modulating the local islet microenvironment and influencing the release of cytotoxic molecules from immune cells. This direct impact on β-cells can lead to dysfunction and apoptosis, which are key hallmarks of T2D progression. The infiltration of immune cells, modulated by RMITRG expression, not only disrupts the β-cell function but also contributes to a chronic inflammatory state within the islets, further exacerbating insulin resistance and β-cell failure (37).

Understanding the intricate relationship between RMITRG expression, immune cell infiltration, and β-cell function is crucial for developing targeted therapies aimed at preserving β-cell function and reducing inflammation in T2D. By targeting the molecular pathways that link RMITRGs to immune cell activity, we may be able to mitigate the detrimental effects of inflammation on β-cells and slow the progression of T2D. This understanding also highlights the potential for therapeutic interventions that could stabilize or even reverse the inflammatory processes contributing to β-cell dysfunction.

Our study revealed that endocrine cells, particularly β cells, engage extensively in cell-cell communication within islets, with macrophages exhibiting the strongest interaction strengths. Information flow analysis identified unique activation of signaling pathways in T2D, including WNT, TGFβ, KIT, LIFR, and HGF, highlighting their roles in disease progression. Notably, the WNT and TGFβ pathways were markedly activated in outgoing signaling from β cells, implicating these pathways in β-cell dysfunction and the broader pathophysiology of T2D.

The WNT pathway, known for its roles in cell fate determination and tissue regeneration, appears to regulate β-cell proliferation and function in T2D (38). While its activation may represent an attempt to compensate for the loss of functional β-cell mass—a hallmark of T2D—chronic activation could lead to β-cell exhaustion and failure due to continuous proliferation without proper differentiation (39). Similarly, the TGFβ pathway, a multifunctional cytokine signaling pathway, inhibits β-cell proliferation and function while promoting extracellular matrix deposition, contributing to islet fibrosis and impaired β-cell functionality (40, 41).

Our receptor-ligand pair analysis further identified Wnt4-(FZD3+LRP6) and TGFB1-(ACVR1B+TGFBR2) as critical mediators of intercellular communication in T2D. These findings underscore the roles of the WNT and TGFβ pathways in β-cell dysfunction and highlight their potential as therapeutic targets for preserving β-cell function and improving outcomes in T2D.

Our study has uncovered distinct expression patterns of DEGs between T2D and non-diabetic patients, shedding light on the molecular signatures that distinguish T2D at the cellular level. The identification of these DEGs, especially those implicated in metal ion transport, provides crucial insights into the dysregulated pathways that contribute to the pathogenesis of T2D. Metal ion transport genes, such as those encoding zinc and iron transporters, play pivotal roles in insulin production, secretion, and activity (13–15). Our findings suggest that disruptions in the normal functioning of these transporters could lead to impaired insulin secretion and action, which are hallmarks of T2D. The dysregulation of such genes not only affects insulin signaling but also influences cellular redox balance and inflammatory responses within the pancreatic islets, potentially exacerbating β-cell dysfunction and insulin resistance.

The biological significance of these DEGs extends to the development of personalized treatment strategies for T2D. The identification of reliable biomarkers among these DEGs could facilitate early diagnosis and patient stratification, enabling more targeted and effective therapeutic interventions. For instance, understanding the specific roles of metal ion transporters in β-cell function could lead to the development of therapies aimed at normalizing their expression or function, thereby improving insulin secretion and overall glycemic control.

In our exploration of the molecular mechanisms underlying T2D, we employed an integrative approach combining scRNA-seq and machine learning to uncover the role of RMITRGs. Moving beyond traditional bulk RNA sequencing or single-gene studies, this approach enabled us to identify a set of hub RMITRGs not previously associated with T2D. These genes, integrated into predictive models, demonstrated high accuracy in distinguishing T2D from non-diabetic states, highlighting their potential as biomarkers and therapeutic targets. Using AlphaFold 3, we predicted the protein structures of these hub RMITRGs, providing new insights into their functional roles and laying the groundwork for future studies.

Our findings also revealed correlations between hub RMITRGs and immune cell infiltration, offering new perspectives on the immunological landscape of T2D. Furthermore, the construction of a PPI network and cell-cell communication analysis provided a systems-level understanding of molecular interactions in T2D, enhancing our knowledge of the disease’s pathophysiology. These insights shed light for targeted and effective treatment strategies, including islet transplantation, which holds promise for restoring endogenous insulin production and improving glycemic control in patients with severe insulin dependence.

However, we acknowledge the limitations of our study. One notable limitation is the inability to predict the structure of the AHNAK gene using AlphaFold 3 due to its large size, which underscores the need for alternative structural biology methods such as cryo-electron microscopy. Additionally, our findings regarding the roles of hub genes in T2D are preliminary, as they lack *in vitro* or clinical validations. The sample size of our study may also introduce potential biases, and the generalizability of our results is yet to be confirmed through larger cohort studies and mechanistic investigations.

Future work with larger cohorts and mechanistic studies is essential for validating our findings and gaining a deeper understanding of the roles of these genes in T2D. Such studies will be crucial for translating our results into clinical practice and for exploring the potential of these genes in diabetes management.

Overall, our study establishes a robust framework for understanding the genetic and immunological underpinnings of T2D. By continuing to investigate these hub genes and their pathways, researchers can advance personalized therapies and optimize clinical outcomes for T2D management.

## Data Availability

The original contributions presented in the study are included in the article/**Supplementary Material**. Further inquiries can be directed to the corresponding authors.

## Supplementary material

Supplementary Figure 1 Upset displaying overlapping genes identified by five algorithms.

Supplementary Figure 2 Correlation analysis between ACTN4, PRNP, TMBIM6, TSPAN13, VMP1, and immune cells.

Supplementary Table 1 Comparison of our cell type annotations with those from the previous study.

Supplementary Table 2 The correlation between 13 hub RMITRGs and immune cells was analyzed in the training sets.

Supplementary Table 3 The correlation between 13 hub RMITRGs and immune cells was analyzed in the testing sets.

Supplementary Table 4 UpSet plot data showing the intersection of genes identified across five analytical algorithms.
