# Supplementary figures and images for: Deciphering the role of metal ion transport-related genes in T2D pathogenesis and immune cell infiltration via scRNA-seq and machine learning
Source: Front Immunol. 2025 Jan 24;15:1479166. doi: 10.3389/fimmu.2024.1479166 (PMC11802808; doi:10.3389/fimmu.2024.1479166)

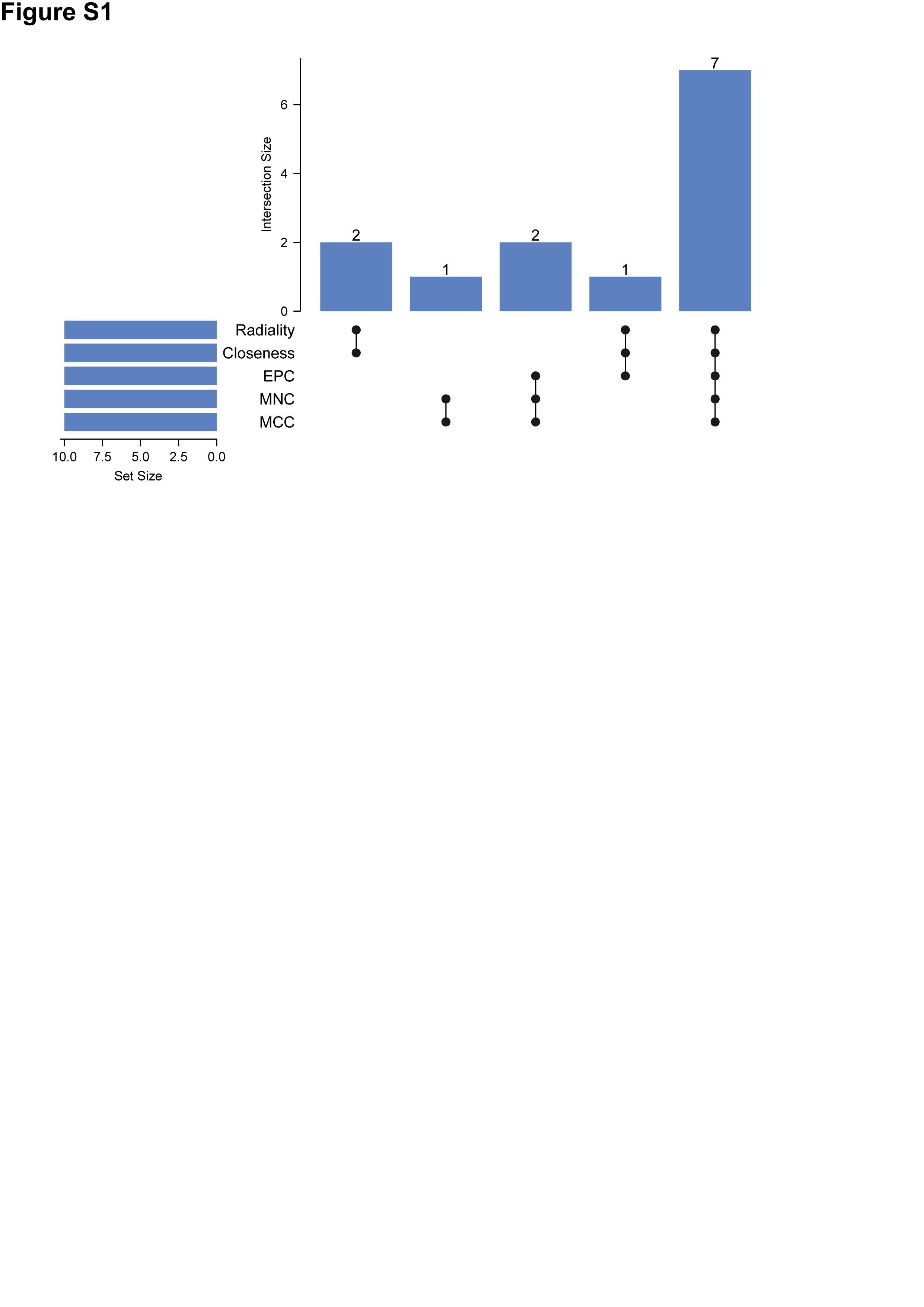

Supplement: Supplementary Figure 1 — Upset displaying overlapping genes identified by five algorithms. [file Image1.tif]

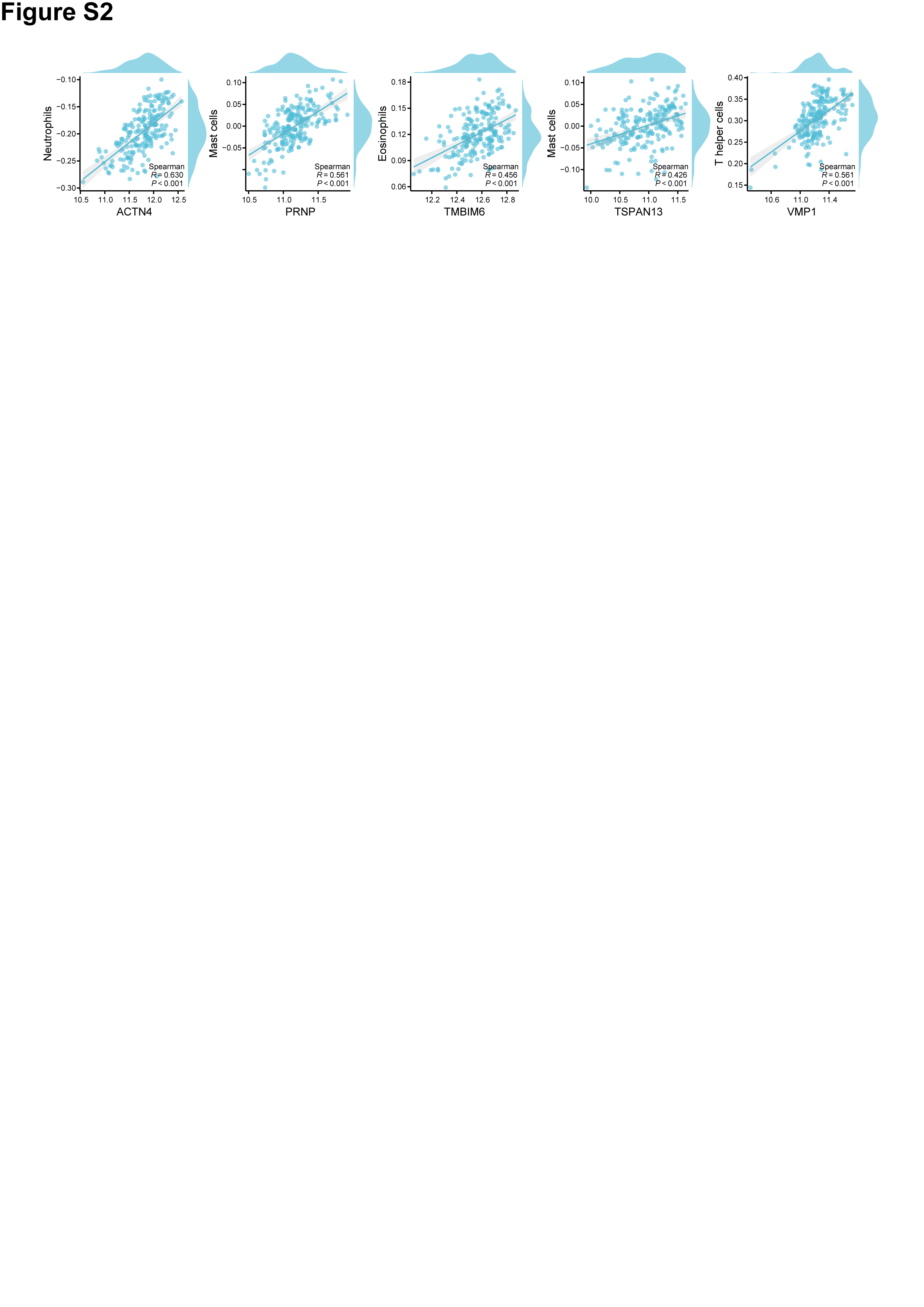

Supplement: Supplementary Figure 2 — Correlation analysis between ACTN4, PRNP, TMBIM6, TSPAN13, VMP1, and immune cells. [file Image2.tif]
